# Dibromidooxido[(*Z*)-*N*′-(propan-2-yl­idene)benzohydrazidato](triphenyl­phosphane)rhenium(V)

**DOI:** 10.1107/S1600536811035811

**Published:** 2011-09-14

**Authors:** Nonzaliseko Yumata, Thomas Gerber, Richard Betz

**Affiliations:** a Nelson Mandela Metropolitan University, Summerstrand Campus, Department of Chemistry, University Way, Summerstrand, PO Box 77000, Port Elizabeth, 6031, South Africa

## Abstract

The asymmetric unit of the title neutral rhenium(V) coordination compound, [ReBr_2_(C_10_H_11_N_2_O)O(C_18_H_15_P)], contains two mol­ecules. In each of the two molecules the metal atom is octa­hedrally coordinated, the bromido ligands being *cis*-orientated. The chelate ligand is present in its imine-tautomeric form. In the crystal, C—H⋯Br contacts connect the mol­ecules into chains along [101]. The shortest inter­centroid distance between two aromatic rings was found to be 3.906 (2) Å.

## Related literature

For the crystal structures of rhenium(I), rhenium(III) and rhenium(V) metal complexes featuring tridentate ON*X* (*X* = O, N, S)-donor Schiff bases, see: Potgieter *et al.* (2010 ▶). For graph-set analysis of hydrogen bonds, see: Etter *et al.* (1990 ▶); Bernstein *et al.* (1995 ▶). For puckering analysis, see: Cremer & Pople (1975 ▶). For general information about radiopharmaceuticals, see: Gerber *et al.* (2011 ▶).
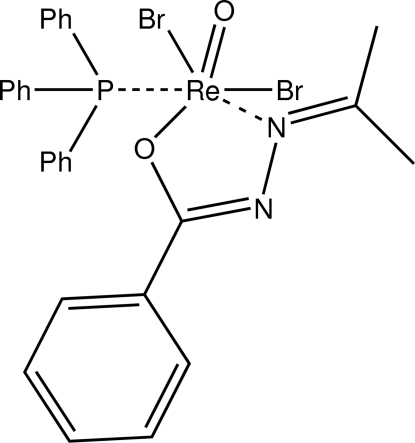


## Experimental

### 

#### Crystal data

[ReBr_2_(C_10_H_11_N_2_O)O(C_18_H_15_P)]
*M* *_r_* = 799.50
Monoclinic, 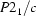

*a* = 19.1130 (5) Å
*b* = 18.3910 (4) Å
*c* = 15.8140 (4) Å
β = 97.410 (1)°
*V* = 5512.3 (2) Å^3^
*Z* = 8
Mo *K*α radiation
μ = 7.40 mm^−1^
*T* = 200 K
0.39 × 0.35 × 0.16 mm

#### Data collection

Bruker APEXII CCD diffractometer
Absorption correction: multi-scan (*SADABS*; Bruker, 2008 ▶) *T*_min_ = 0.589, *T*_max_ = 1.000
50272 measured reflections
13695 independent reflections
11062 reflections with *I* > 2σ(*I*)
*R*_int_ = 0.031

#### Refinement

*R*[*F* ^2^ > 2σ(*F* ^2^)] = 0.028
*wR*(*F* ^2^) = 0.066
*S* = 1.11
13695 reflections
649 parameters
H-atom parameters constrained
Δρ_max_ = 1.11 e Å^−3^
Δρ_min_ = −1.33 e Å^−3^

### 

Data collection: *APEX2* (Bruker, 2010 ▶); cell refinement: *SAINT* (Bruker, 2010 ▶); data reduction: *SAINT*; program(s) used to solve structure: *SIR97* (Altomare *et al.*, 1999 ▶); program(s) used to refine structure: *SHELXL97* (Sheldrick, 2008 ▶); molecular graphics: *ORTEPIII* (Farrugia, 1997 ▶) and *Mercury* (Macrae *et al.*, 2008 ▶); software used to prepare material for publication: *SHELXL97* and *PLATON* (Spek, 2009 ▶).

## Supplementary Material

Crystal structure: contains datablock(s) I, global. DOI: 10.1107/S1600536811035811/ff2026sup1.cif

Supplementary material file. DOI: 10.1107/S1600536811035811/ff2026Isup2.cdx

Structure factors: contains datablock(s) I. DOI: 10.1107/S1600536811035811/ff2026Isup3.hkl

Additional supplementary materials:  crystallographic information; 3D view; checkCIF report

## Figures and Tables

**Table 1:** Hydrogen-bond geometry (Å, °)

*D*—H⋯*A*	*D*—H	H⋯*A*	*D*⋯*A*	*D*—H⋯*A*
C22—H22⋯Br3	0.95	2.94	3.819 (5)	154
C85—H85⋯Br2^i^	0.95	2.92	3.587 (4)	128

Symmetry code: (i) 
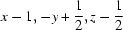
.

**Fig. 1.**
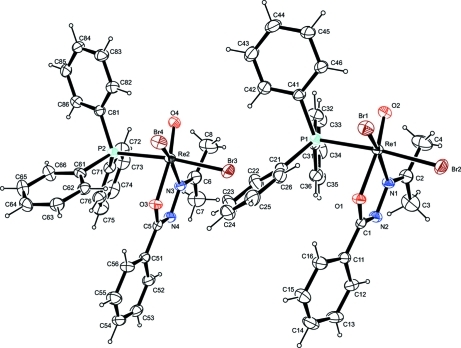
The molecular structure of the title compound, with atom labels and anisotropic displacement ellipsoids (drawn at 50% probability level).

**Fig. 2.**
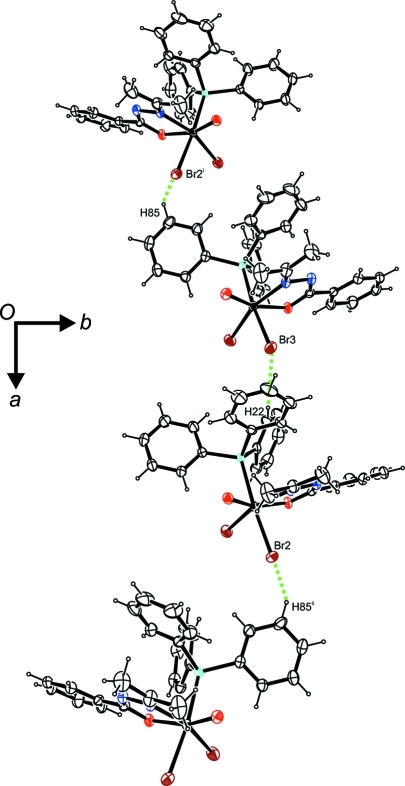
Intermolecular contacts, viewed along [0 0 - 1]. Symmetry operators: ix - 1, -y + 1/2, z - 1/2; iix + 1, -y + 1/2, z + 1/2.

## supplementary crystallographic information

### Comment

Next to cardiovascular diseases, cancer has become one of the main fatal diseases in industrialized countries. Apart from classical surgery, chemo- and radiotherapeutic treatments have entered the arsenal of possible cures for certain types of cancer. All methods, however, suffer from their own set of problematic side-effects and, as a consequence, the development of radiopharmaceuticals – combining the advantages of chemotherapy as well as radiation methods while at the same time avoiding their unique respective undesired side-effects – has been a topic of research (Gerber *et al.*, 2011). Tailoring and fine-tuning of the envisioned radiopharmaceuticals' properties such as lipophilicity and, in particular, inertness is of paramount importance with respect to possible future *in vivo* applications in contemporary medicine and requires sound knowledge about structural parameters of the ligands applied if a more heuristic approach in the synthesis is to triumph over pure trial-and-error as it is encountered in this specific field of coordination chemistry up to the present day. In continuation of our interest in rhenium-based coordination compounds that might serve as radiopharmaceuticals, we determined the molecular and crystal structure of the title compound. Information about the crystal structures of rhenium coordination compounds with the central atom in different oxidation states is apparent in the literature (Potgieter *et al.*, 2010).

The central atom in both molecules of the asymmetric unit is hexacoordinate. The ligand sphere features one chelate ligand which is present in its tautomeric imine form. Both oxygen atoms are in *trans*-position, the bromido ligands are *cis*-orientated. While the small puckering amplitude of one of the five-membered chelate rings (τ = 4.3 °) precludes a conformational analysis (Cremer & Pople, 1975) in the first molecule of the asymmetric unite, the corresponding five-membered ring in the second molecule adopts an *envelope* conformation on the rhenium atom (^Re2^*E*, *Q*_2_ = 0.076 (3) Å, π_2_ = 358 (3) °). The central atom is displaced by 0.184 (1) Å and 0.188 (1) Å, respectively, from the plane defined by the bromido ligands as well as the coordinating nitrogen atom and phosphorus atom (Fig. 1).

In the crystal, C–H···Br contacts can be observed whose range falls by more than 0.1 Å below the sum of van-der-Waals radii of the respective atoms. These are supported exclusively by hydrogen atoms of phenyl groups on the triphenylphosphane ligand and involve hydrogen atoms in *ortho* as well as in *meta* position as donors and the bromido ligand in *trans*-position to the coordinating phosphorus atom as acceptors. In terms of graph-set analysis (Etter *et al.*, 1990; Bernstein *et al.*, 1995), the descriptor for these contacts is *DD* on the unitary level. In total, the molecules are connected to chains along [1 0 1] (Fig. 2). The shortest intercentroid distance between two centers of gravity was found at 3.906 (2) Å.

### Experimental

Benzhydrazide and *trans*-[ReOBr_3_(PPh_3_)_2_] were refluxed in acetone for 5 h. After cooling to room temperature and filtration the filtrate was stored at room temperature upon which crystals formed in the course of a couple of days.

### Refinement

Carbon-bound H atoms were placed in calculated positions (C—H 0.95 Å for aromatic C atoms, C—H 0.98 Å for methyl groups) and were included in the refinement in the riding model approximation, with *U*(H) set to 1.2*U*_eq_(C) for aromatic carbon atoms and *U*(H) set to 1.5*U*_eq_(C) for methyl groups.

### Crystal data

**Table:**

[ReBr_2_(C_10_H_11_N_2_O)O(C_18_H_15_P)]	*F*(000) = 3072
*M**_r_* = 799.50	*D*_x_ = 1.927 Mg m^−^^3^
Monoclinic, *P*2_1_/*c*	Mo *K*α radiation, λ = 0.71069 Å
Hall symbol: -P 2ybc	Cell parameters from 9749 reflections
*a* = 19.1130 (5) Å	θ = 2.9–28.3°
*b* = 18.3910 (4) Å	µ = 7.40 mm^−^^1^
*c* = 15.8140 (4) Å	*T* = 200 K
β = 97.410 (1)°	Platelet, green
*V* = 5512.3 (2) Å^3^	0.39 × 0.35 × 0.16 mm
*Z* = 8	

### Data collection

**Table:**

Bruker APEXII CCD diffractometer	13695 independent reflections
Radiation source: fine-focus sealed tube	11062 reflections with *I* > 2σ(*I*)
graphite	*R*_int_ = 0.031
φ and ω scans	θ_max_ = 28.3°, θ_min_ = 1.5°
Absorption correction: multi-scan (*SADABS*; Bruker, 2008)	*h* = −25→25
*T*_min_ = 0.589, *T*_max_ = 1.000	*k* = −24→20
50272 measured reflections	*l* = −21→20

### Refinement

**Table:**

Refinement on *F*^2^	Primary atom site location: structure-invariant direct methods
Least-squares matrix: full	Secondary atom site location: difference Fourier map
*R*[*F*^2^ > 2σ(*F*^2^)] = 0.028	Hydrogen site location: inferred from neighbouring sites
*wR*(*F*^2^) = 0.066	H-atom parameters constrained
*S* = 1.11	*w* = 1/[σ^2^(*F*_o_^2^) + (0.0171*P*)^2^ + 13.1603*P*] where *P* = (*F*_o_^2^ + 2*F*_c_^2^)/3
13695 reflections	(Δ/σ)_max_ = 0.003
649 parameters	Δρ_max_ = 1.11 e Å^−^^3^
0 restraints	Δρ_min_ = −1.33 e Å^−^^3^

### Fractional atomic coordinates and isotropic or equivalent isotropic displacement parameters (Å^2^)

**Table:**

	*x*	*y*	*z*	*U*_iso_*/*U*_eq_	
Re1	0.735701 (7)	0.329200 (8)	0.354383 (9)	0.01810 (4)	
Br1	0.79252 (2)	0.26176 (2)	0.24550 (3)	0.02955 (9)	
Br2	0.85659 (2)	0.37295 (2)	0.41703 (3)	0.03182 (9)	
P1	0.61621 (5)	0.29731 (5)	0.28519 (6)	0.01951 (19)	
O1	0.72398 (14)	0.41638 (13)	0.27545 (15)	0.0217 (5)	
O2	0.71940 (14)	0.26514 (14)	0.42482 (16)	0.0255 (6)	
N1	0.69989 (17)	0.41714 (16)	0.42803 (19)	0.0214 (6)	
N2	0.68319 (17)	0.48183 (17)	0.3824 (2)	0.0241 (7)	
C1	0.69745 (19)	0.47580 (19)	0.3050 (2)	0.0201 (7)	
C2	0.6947 (2)	0.4213 (2)	0.5092 (2)	0.0289 (9)	
C3	0.6707 (3)	0.4897 (3)	0.5471 (3)	0.0453 (12)	
H311	0.7091	0.5254	0.5522	0.068*	
H321	0.6575	0.4794	0.6037	0.068*	
H331	0.6299	0.5093	0.5103	0.068*	
C4	0.7118 (3)	0.3590 (3)	0.5684 (3)	0.0494 (14)	
H411	0.6718	0.3252	0.5635	0.074*	
H421	0.7212	0.3770	0.6271	0.074*	
H431	0.7537	0.3338	0.5534	0.074*	
C11	0.68113 (19)	0.5372 (2)	0.2456 (2)	0.0218 (8)	
C12	0.6662 (2)	0.6056 (2)	0.2763 (3)	0.0278 (9)	
H12	0.6682	0.6135	0.3359	0.033*	
C13	0.6484 (2)	0.6622 (2)	0.2198 (3)	0.0335 (10)	
H13	0.6379	0.7089	0.2407	0.040*	
C14	0.6458 (2)	0.6509 (2)	0.1333 (3)	0.0356 (10)	
H14	0.6338	0.6899	0.0948	0.043*	
C15	0.6607 (2)	0.5832 (2)	0.1023 (3)	0.0362 (10)	
H15	0.6587	0.5757	0.0426	0.043*	
C16	0.6784 (2)	0.5264 (2)	0.1581 (3)	0.0289 (9)	
H16	0.6888	0.4799	0.1368	0.035*	
C21	0.5865 (2)	0.3391 (2)	0.1828 (2)	0.0237 (8)	
C22	0.5194 (2)	0.3683 (3)	0.1637 (3)	0.0364 (10)	
H22	0.4877	0.3677	0.2053	0.044*	
C23	0.4984 (3)	0.3984 (3)	0.0841 (3)	0.0475 (13)	
H23	0.4525	0.4187	0.0714	0.057*	
C24	0.5439 (3)	0.3990 (3)	0.0233 (3)	0.0439 (12)	
H24	0.5296	0.4203	−0.0309	0.053*	
C25	0.6101 (3)	0.3688 (3)	0.0409 (3)	0.0394 (11)	
H25	0.6411	0.3686	−0.0015	0.047*	
C26	0.6317 (2)	0.3389 (2)	0.1204 (3)	0.0295 (9)	
H26	0.6775	0.3182	0.1324	0.035*	
C31	0.5533 (2)	0.3228 (2)	0.3572 (2)	0.0235 (8)	
C32	0.5277 (2)	0.2710 (2)	0.4097 (3)	0.0333 (10)	
H32	0.5390	0.2211	0.4036	0.040*	
C33	0.4857 (3)	0.2918 (3)	0.4709 (3)	0.0430 (12)	
H33	0.4689	0.2564	0.5071	0.052*	
C34	0.4686 (3)	0.3640 (3)	0.4792 (3)	0.0429 (12)	
H34	0.4394	0.3782	0.5205	0.051*	
C35	0.4938 (2)	0.4158 (3)	0.4274 (3)	0.0376 (10)	
H35	0.4819	0.4655	0.4334	0.045*	
C36	0.5362 (2)	0.3956 (2)	0.3673 (3)	0.0288 (9)	
H36	0.5538	0.4316	0.3324	0.035*	
C41	0.6015 (2)	0.2002 (2)	0.2683 (2)	0.0224 (8)	
C42	0.5376 (2)	0.1775 (2)	0.2246 (3)	0.0363 (10)	
H42	0.5043	0.2126	0.2007	0.044*	
C43	0.5220 (2)	0.1044 (2)	0.2156 (3)	0.0425 (12)	
H43	0.4781	0.0895	0.1854	0.051*	
C44	0.5698 (2)	0.0531 (2)	0.2502 (3)	0.0371 (10)	
H44	0.5587	0.0028	0.2445	0.045*	
C45	0.6337 (2)	0.0747 (2)	0.2931 (3)	0.0348 (10)	
H45	0.6668	0.0392	0.3164	0.042*	
C46	0.6499 (2)	0.1481 (2)	0.3026 (3)	0.0281 (9)	
H46	0.6940	0.1628	0.3323	0.034*	
Re2	0.234120 (8)	0.330538 (8)	0.174643 (9)	0.01962 (4)	
Br3	0.34651 (2)	0.37327 (2)	0.26052 (3)	0.03643 (10)	
Br4	0.30700 (2)	0.27118 (2)	0.07404 (3)	0.03180 (9)	
P2	0.12037 (5)	0.30358 (5)	0.08588 (6)	0.01950 (19)	
O3	0.22884 (14)	0.42150 (13)	0.10244 (16)	0.0233 (6)	
O4	0.21229 (15)	0.26075 (15)	0.23405 (16)	0.0263 (6)	
N3	0.18502 (17)	0.41223 (17)	0.24467 (19)	0.0233 (7)	
N4	0.17059 (18)	0.47831 (17)	0.20169 (19)	0.0246 (7)	
C5	0.19517 (19)	0.4780 (2)	0.1291 (2)	0.0208 (7)	
C6	0.1668 (2)	0.4106 (2)	0.3211 (3)	0.0335 (10)	
C7	0.1285 (3)	0.4726 (3)	0.3551 (3)	0.0473 (13)	
H711	0.0797	0.4582	0.3592	0.071*	
H721	0.1288	0.5143	0.3166	0.071*	
H731	0.1520	0.4860	0.4117	0.071*	
C8	0.1822 (3)	0.3458 (3)	0.3778 (3)	0.0515 (14)	
H811	0.2286	0.3260	0.3701	0.077*	
H821	0.1459	0.3087	0.3629	0.077*	
H831	0.1822	0.3604	0.4374	0.077*	
C51	0.1850 (2)	0.5422 (2)	0.0732 (2)	0.0227 (8)	
C52	0.1554 (2)	0.6056 (2)	0.1016 (3)	0.0281 (9)	
H52	0.1420	0.6076	0.1573	0.034*	
C53	0.1455 (2)	0.6653 (2)	0.0489 (3)	0.0318 (9)	
H53	0.1259	0.7086	0.0687	0.038*	
C54	0.1640 (2)	0.6627 (2)	−0.0328 (3)	0.0337 (10)	
H54	0.1565	0.7039	−0.0692	0.040*	
C55	0.1935 (2)	0.6000 (2)	−0.0616 (3)	0.0357 (10)	
H55	0.2064	0.5981	−0.1176	0.043*	
C56	0.2042 (2)	0.5402 (2)	−0.0087 (3)	0.0284 (9)	
H56	0.2248	0.4974	−0.0283	0.034*	
C61	0.1077 (2)	0.3279 (2)	−0.0267 (2)	0.0229 (8)	
C62	0.1628 (2)	0.3508 (2)	−0.0694 (2)	0.0258 (8)	
H62	0.2091	0.3548	−0.0397	0.031*	
C63	0.1504 (2)	0.3678 (2)	−0.1550 (2)	0.0308 (9)	
H63	0.1883	0.3834	−0.1840	0.037*	
C64	0.0835 (2)	0.3623 (2)	−0.1987 (2)	0.0334 (10)	
H64	0.0751	0.3746	−0.2574	0.040*	
C65	0.0284 (2)	0.3389 (3)	−0.1569 (3)	0.0367 (10)	
H65	−0.0177	0.3345	−0.1871	0.044*	
C66	0.0404 (2)	0.3218 (2)	−0.0714 (3)	0.0330 (9)	
H66	0.0024	0.3058	−0.0428	0.040*	
C71	0.0498 (2)	0.3505 (2)	0.1299 (2)	0.0241 (8)	
C72	0.0273 (2)	0.3236 (2)	0.2048 (3)	0.0346 (10)	
H72	0.0458	0.2789	0.2281	0.042*	
C73	−0.0212 (3)	0.3611 (3)	0.2450 (3)	0.0460 (12)	
H73	−0.0368	0.3421	0.2952	0.055*	
C74	−0.0471 (3)	0.4268 (3)	0.2117 (3)	0.0491 (13)	
H74	−0.0806	0.4530	0.2392	0.059*	
C75	−0.0246 (3)	0.4544 (3)	0.1392 (3)	0.0445 (12)	
H75	−0.0424	0.4998	0.1172	0.053*	
C76	0.0236 (2)	0.4168 (2)	0.0980 (3)	0.0323 (9)	
H76	0.0389	0.4363	0.0478	0.039*	
C81	0.0972 (2)	0.2071 (2)	0.0871 (2)	0.0224 (8)	
C82	0.1500 (2)	0.1548 (2)	0.0970 (2)	0.0261 (8)	
H82	0.1981	0.1693	0.1058	0.031*	
C83	0.1329 (2)	0.0814 (2)	0.0944 (3)	0.0309 (9)	
H83	0.1694	0.0460	0.1010	0.037*	
C84	0.0632 (2)	0.0598 (2)	0.0822 (3)	0.0329 (10)	
H84	0.0516	0.0096	0.0806	0.039*	
C85	0.0101 (2)	0.1115 (2)	0.0723 (3)	0.0354 (10)	
H85	−0.0379	0.0967	0.0637	0.043*	
C86	0.0270 (2)	0.1849 (2)	0.0749 (3)	0.0311 (9)	
H86	−0.0096	0.2202	0.0684	0.037*	

### Atomic displacement parameters (Å^2^)

**Table:**

	*U*^11^	*U*^22^	*U*^33^	*U*^12^	*U*^13^	*U*^23^
Re1	0.01965 (7)	0.01528 (7)	0.01927 (7)	0.00080 (6)	0.00211 (5)	0.00075 (5)
Br1	0.0336 (2)	0.0283 (2)	0.02781 (19)	0.00461 (17)	0.00804 (16)	−0.00207 (16)
Br2	0.0251 (2)	0.0300 (2)	0.0393 (2)	−0.00504 (17)	0.00004 (17)	−0.00410 (18)
P1	0.0186 (5)	0.0168 (4)	0.0226 (4)	0.0018 (4)	0.0007 (4)	0.0004 (4)
O1	0.0285 (14)	0.0158 (12)	0.0210 (12)	0.0032 (11)	0.0046 (11)	0.0015 (10)
O2	0.0267 (14)	0.0231 (14)	0.0263 (14)	0.0032 (11)	0.0024 (11)	0.0039 (11)
N1	0.0257 (17)	0.0173 (15)	0.0213 (15)	0.0032 (13)	0.0032 (13)	−0.0004 (12)
N2	0.0287 (17)	0.0183 (16)	0.0248 (16)	0.0028 (14)	0.0014 (13)	0.0013 (13)
C1	0.0172 (17)	0.0178 (18)	0.0246 (18)	−0.0031 (14)	−0.0002 (14)	−0.0013 (14)
C2	0.033 (2)	0.029 (2)	0.0251 (19)	0.0011 (18)	0.0053 (17)	−0.0027 (16)
C3	0.062 (3)	0.045 (3)	0.030 (2)	0.016 (3)	0.011 (2)	−0.010 (2)
C4	0.083 (4)	0.041 (3)	0.026 (2)	0.014 (3)	0.012 (2)	0.008 (2)
C11	0.0202 (18)	0.0181 (18)	0.0271 (19)	0.0004 (15)	0.0032 (15)	0.0023 (15)
C12	0.031 (2)	0.0198 (19)	0.033 (2)	−0.0027 (17)	0.0050 (17)	0.0001 (16)
C13	0.033 (2)	0.019 (2)	0.049 (3)	0.0045 (17)	0.009 (2)	0.0052 (18)
C14	0.032 (2)	0.032 (2)	0.043 (2)	0.0039 (19)	0.0039 (19)	0.018 (2)
C15	0.041 (3)	0.035 (2)	0.032 (2)	0.002 (2)	0.0011 (19)	0.0102 (19)
C16	0.035 (2)	0.024 (2)	0.028 (2)	0.0020 (17)	0.0047 (17)	0.0019 (16)
C21	0.029 (2)	0.0169 (18)	0.0239 (18)	0.0008 (15)	−0.0010 (15)	0.0008 (14)
C22	0.034 (2)	0.040 (3)	0.034 (2)	0.009 (2)	−0.0013 (19)	0.000 (2)
C23	0.050 (3)	0.048 (3)	0.039 (3)	0.019 (3)	−0.013 (2)	0.003 (2)
C24	0.066 (4)	0.033 (2)	0.028 (2)	0.000 (2)	−0.011 (2)	0.0034 (19)
C25	0.055 (3)	0.040 (3)	0.022 (2)	−0.013 (2)	0.001 (2)	−0.0006 (18)
C26	0.031 (2)	0.030 (2)	0.027 (2)	−0.0029 (18)	0.0010 (16)	−0.0032 (17)
C31	0.0187 (18)	0.025 (2)	0.0259 (19)	0.0029 (16)	0.0003 (15)	−0.0008 (15)
C32	0.037 (2)	0.030 (2)	0.034 (2)	0.0067 (19)	0.0094 (19)	0.0039 (18)
C33	0.044 (3)	0.052 (3)	0.035 (2)	0.006 (2)	0.015 (2)	0.006 (2)
C34	0.038 (3)	0.062 (3)	0.030 (2)	0.015 (2)	0.010 (2)	−0.007 (2)
C35	0.032 (2)	0.037 (3)	0.044 (3)	0.010 (2)	0.003 (2)	−0.011 (2)
C36	0.024 (2)	0.025 (2)	0.037 (2)	0.0036 (17)	0.0036 (17)	−0.0046 (17)
C41	0.0214 (19)	0.0181 (18)	0.0279 (19)	0.0010 (15)	0.0042 (15)	−0.0020 (15)
C42	0.032 (2)	0.025 (2)	0.048 (3)	0.0042 (19)	−0.009 (2)	−0.0017 (19)
C43	0.031 (2)	0.030 (2)	0.063 (3)	−0.004 (2)	−0.008 (2)	−0.009 (2)
C44	0.039 (3)	0.020 (2)	0.052 (3)	−0.0037 (19)	0.002 (2)	−0.0067 (19)
C45	0.032 (2)	0.021 (2)	0.050 (3)	0.0038 (18)	−0.003 (2)	−0.0011 (19)
C46	0.026 (2)	0.0202 (19)	0.037 (2)	−0.0003 (16)	0.0000 (17)	−0.0027 (17)
Re2	0.02176 (8)	0.01789 (7)	0.01966 (7)	−0.00075 (6)	0.00433 (5)	0.00207 (5)
Br3	0.0303 (2)	0.0309 (2)	0.0458 (2)	−0.00655 (18)	−0.00375 (18)	−0.00186 (19)
Br4	0.0334 (2)	0.0324 (2)	0.0322 (2)	0.00594 (18)	0.01408 (17)	0.00429 (17)
P2	0.0210 (5)	0.0188 (4)	0.0194 (4)	0.0006 (4)	0.0053 (4)	0.0011 (4)
O3	0.0292 (14)	0.0176 (13)	0.0243 (13)	0.0014 (11)	0.0077 (11)	0.0033 (10)
O4	0.0303 (15)	0.0265 (14)	0.0219 (13)	−0.0014 (12)	0.0025 (11)	0.0034 (11)
N3	0.0295 (17)	0.0184 (16)	0.0220 (15)	−0.0019 (13)	0.0039 (13)	−0.0001 (12)
N4	0.0323 (18)	0.0196 (16)	0.0221 (15)	0.0000 (14)	0.0041 (13)	0.0003 (13)
C5	0.0195 (18)	0.0201 (18)	0.0218 (17)	−0.0039 (15)	−0.0011 (14)	−0.0022 (14)
C6	0.047 (3)	0.033 (2)	0.0221 (19)	−0.002 (2)	0.0112 (18)	−0.0043 (17)
C7	0.067 (4)	0.047 (3)	0.033 (2)	0.004 (3)	0.025 (2)	−0.007 (2)
C8	0.092 (4)	0.044 (3)	0.022 (2)	0.006 (3)	0.020 (2)	0.006 (2)
C51	0.0203 (18)	0.0203 (19)	0.0267 (19)	−0.0035 (15)	0.0005 (15)	0.0024 (15)
C52	0.027 (2)	0.023 (2)	0.034 (2)	−0.0011 (17)	0.0030 (17)	−0.0008 (17)
C53	0.026 (2)	0.020 (2)	0.047 (3)	0.0011 (17)	−0.0019 (18)	−0.0033 (18)
C54	0.032 (2)	0.023 (2)	0.043 (2)	−0.0047 (18)	−0.0029 (19)	0.0092 (18)
C55	0.043 (3)	0.033 (2)	0.032 (2)	−0.002 (2)	0.009 (2)	0.0068 (18)
C56	0.034 (2)	0.022 (2)	0.031 (2)	−0.0009 (17)	0.0071 (17)	0.0054 (16)
C61	0.030 (2)	0.0203 (18)	0.0189 (17)	0.0047 (16)	0.0056 (15)	0.0025 (14)
C62	0.031 (2)	0.0233 (19)	0.0238 (19)	−0.0021 (17)	0.0065 (16)	−0.0019 (15)
C63	0.042 (2)	0.029 (2)	0.0227 (19)	−0.0019 (19)	0.0104 (18)	−0.0005 (16)
C64	0.051 (3)	0.033 (2)	0.0165 (18)	0.008 (2)	0.0038 (18)	−0.0013 (16)
C65	0.032 (2)	0.050 (3)	0.026 (2)	0.006 (2)	−0.0031 (17)	−0.0018 (19)
C66	0.027 (2)	0.044 (3)	0.027 (2)	0.0034 (19)	0.0041 (17)	0.0043 (18)
C71	0.0231 (19)	0.0240 (19)	0.0263 (19)	−0.0009 (16)	0.0074 (15)	−0.0044 (15)
C72	0.035 (2)	0.036 (2)	0.035 (2)	0.000 (2)	0.0121 (19)	0.0014 (19)
C73	0.037 (3)	0.065 (4)	0.039 (3)	−0.002 (2)	0.017 (2)	−0.009 (2)
C74	0.035 (3)	0.062 (4)	0.052 (3)	0.008 (2)	0.014 (2)	−0.025 (3)
C75	0.039 (3)	0.034 (3)	0.060 (3)	0.014 (2)	0.004 (2)	−0.014 (2)
C76	0.033 (2)	0.027 (2)	0.037 (2)	0.0004 (18)	0.0050 (18)	−0.0038 (18)
C81	0.029 (2)	0.0206 (19)	0.0178 (17)	−0.0019 (16)	0.0034 (15)	−0.0007 (14)
C82	0.030 (2)	0.024 (2)	0.0255 (19)	0.0008 (16)	0.0079 (16)	−0.0010 (15)
C83	0.041 (2)	0.024 (2)	0.029 (2)	0.0032 (18)	0.0068 (18)	−0.0031 (16)
C84	0.049 (3)	0.022 (2)	0.026 (2)	−0.0059 (19)	−0.0021 (19)	−0.0004 (16)
C85	0.032 (2)	0.034 (2)	0.037 (2)	−0.0116 (19)	−0.0091 (18)	0.0049 (19)
C86	0.031 (2)	0.026 (2)	0.034 (2)	−0.0006 (18)	−0.0034 (18)	0.0038 (17)

### Geometric parameters (Å, °)

**Table:**

Re1—O2	1.678 (3)	Re2—O4	1.675 (3)
Re1—O1	2.027 (2)	Re2—O3	2.021 (2)
Re1—N1	2.155 (3)	Re2—N3	2.152 (3)
Re1—P1	2.4724 (10)	Re2—P2	2.4810 (10)
Re1—Br1	2.4829 (4)	Re2—Br4	2.4970 (4)
Re1—Br2	2.5252 (4)	Re2—Br3	2.5147 (5)
P1—C21	1.816 (4)	P2—C71	1.814 (4)
P1—C31	1.821 (4)	P2—C61	1.821 (4)
P1—C41	1.821 (4)	P2—C81	1.830 (4)
O1—C1	1.315 (4)	O3—C5	1.320 (4)
N1—C2	1.303 (5)	N3—C6	1.301 (5)
N1—N2	1.406 (4)	N3—N4	1.402 (4)
N2—C1	1.294 (5)	N4—C5	1.295 (5)
C1—C11	1.476 (5)	C5—C51	1.473 (5)
C2—C4	1.489 (6)	C6—C7	1.491 (6)
C2—C3	1.491 (6)	C6—C8	1.496 (6)
C3—H311	0.9800	C7—H711	0.9800
C3—H321	0.9800	C7—H721	0.9800
C3—H331	0.9800	C7—H731	0.9800
C4—H411	0.9800	C8—H811	0.9800
C4—H421	0.9800	C8—H821	0.9800
C4—H431	0.9800	C8—H831	0.9800
C11—C12	1.391 (5)	C51—C56	1.392 (5)
C11—C16	1.393 (5)	C51—C52	1.395 (5)
C12—C13	1.385 (6)	C52—C53	1.377 (6)
C12—H12	0.9500	C52—H52	0.9500
C13—C14	1.378 (6)	C53—C54	1.384 (6)
C13—H13	0.9500	C53—H53	0.9500
C14—C15	1.381 (6)	C54—C55	1.387 (6)
C14—H14	0.9500	C54—H54	0.9500
C15—C16	1.382 (6)	C55—C56	1.380 (6)
C15—H15	0.9500	C55—H55	0.9500
C16—H16	0.9500	C56—H56	0.9500
C21—C22	1.387 (6)	C61—C62	1.387 (5)
C21—C26	1.393 (6)	C61—C66	1.391 (6)
C22—C23	1.386 (6)	C62—C63	1.380 (5)
C22—H22	0.9500	C62—H62	0.9500
C23—C24	1.378 (7)	C63—C64	1.377 (6)
C23—H23	0.9500	C63—H63	0.9500
C24—C25	1.377 (7)	C64—C65	1.382 (6)
C24—H24	0.9500	C64—H64	0.9500
C25—C26	1.386 (6)	C65—C66	1.378 (6)
C25—H25	0.9500	C65—H65	0.9500
C26—H26	0.9500	C66—H66	0.9500
C31—C36	1.393 (5)	C71—C76	1.388 (6)
C31—C32	1.395 (6)	C71—C72	1.401 (5)
C32—C33	1.388 (6)	C72—C73	1.376 (6)
C32—H32	0.9500	C72—H72	0.9500
C33—C34	1.376 (7)	C73—C74	1.383 (8)
C33—H33	0.9500	C73—H73	0.9500
C34—C35	1.383 (7)	C74—C75	1.373 (7)
C34—H34	0.9500	C74—H74	0.9500
C35—C36	1.378 (6)	C75—C76	1.382 (6)
C35—H35	0.9500	C75—H75	0.9500
C36—H36	0.9500	C76—H76	0.9500
C41—C42	1.388 (6)	C81—C82	1.389 (5)
C41—C46	1.392 (5)	C81—C86	1.391 (6)
C42—C43	1.381 (6)	C82—C83	1.387 (6)
C42—H42	0.9500	C82—H82	0.9500
C43—C44	1.377 (6)	C83—C84	1.381 (6)
C43—H43	0.9500	C83—H83	0.9500
C44—C45	1.376 (6)	C84—C85	1.384 (6)
C44—H44	0.9500	C84—H84	0.9500
C45—C46	1.389 (6)	C85—C86	1.388 (6)
C45—H45	0.9500	C85—H85	0.9500
C46—H46	0.9500	C86—H86	0.9500
			
O2—Re1—O1	161.65 (12)	O4—Re2—O3	162.46 (12)
O2—Re1—N1	93.96 (12)	O4—Re2—N3	95.14 (12)
O1—Re1—N1	73.84 (10)	O3—Re2—N3	73.71 (11)
O2—Re1—P1	83.53 (10)	O4—Re2—P2	84.30 (10)
O1—Re1—P1	83.83 (8)	O3—Re2—P2	82.50 (8)
N1—Re1—P1	94.44 (9)	N3—Re2—P2	91.27 (9)
O2—Re1—Br1	104.18 (9)	O4—Re2—Br4	102.84 (9)
O1—Re1—Br1	89.52 (7)	O3—Re2—Br4	89.61 (7)
N1—Re1—Br1	161.31 (8)	N3—Re2—Br4	161.64 (8)
P1—Re1—Br1	92.08 (2)	P2—Re2—Br4	94.15 (2)
O2—Re1—Br2	101.66 (9)	O4—Re2—Br3	101.18 (9)
O1—Re1—Br2	90.71 (8)	O3—Re2—Br3	91.34 (8)
N1—Re1—Br2	83.52 (9)	N3—Re2—Br3	84.42 (9)
P1—Re1—Br2	174.53 (2)	P2—Re2—Br3	173.29 (3)
Br1—Re1—Br2	88.359 (15)	Br4—Re2—Br3	88.444 (16)
C21—P1—C31	107.05 (18)	C71—P2—C61	104.28 (18)
C21—P1—C41	105.04 (17)	C71—P2—C81	105.30 (18)
C31—P1—C41	104.00 (17)	C61—P2—C81	104.29 (17)
C21—P1—Re1	117.33 (13)	C71—P2—Re2	109.17 (13)
C31—P1—Re1	107.98 (13)	C61—P2—Re2	120.00 (13)
C41—P1—Re1	114.44 (13)	C81—P2—Re2	112.58 (13)
C1—O1—Re1	117.1 (2)	C5—O3—Re2	117.5 (2)
C2—N1—N2	114.5 (3)	C6—N3—N4	114.3 (3)
C2—N1—Re1	130.3 (3)	C6—N3—Re2	130.4 (3)
N2—N1—Re1	115.1 (2)	N4—N3—Re2	115.3 (2)
C1—N2—N1	110.6 (3)	C5—N4—N3	110.8 (3)
N2—C1—O1	123.0 (3)	N4—C5—O3	122.3 (3)
N2—C1—C11	118.9 (3)	N4—C5—C51	119.6 (3)
O1—C1—C11	118.0 (3)	O3—C5—C51	118.2 (3)
N1—C2—C4	122.4 (4)	N3—C6—C7	121.3 (4)
N1—C2—C3	120.8 (4)	N3—C6—C8	121.3 (4)
C4—C2—C3	116.8 (4)	C7—C6—C8	117.5 (4)
C2—C3—H311	109.5	C6—C7—H711	109.5
C2—C3—H321	109.5	C6—C7—H721	109.5
H311—C3—H321	109.5	H711—C7—H721	109.5
C2—C3—H331	109.5	C6—C7—H731	109.5
H311—C3—H331	109.5	H711—C7—H731	109.5
H321—C3—H331	109.5	H721—C7—H731	109.5
C2—C4—H411	109.5	C6—C8—H811	109.5
C2—C4—H421	109.5	C6—C8—H821	109.5
H411—C4—H421	109.5	H811—C8—H821	109.5
C2—C4—H431	109.5	C6—C8—H831	109.5
H411—C4—H431	109.5	H811—C8—H831	109.5
H421—C4—H431	109.5	H821—C8—H831	109.5
C12—C11—C16	119.5 (4)	C56—C51—C52	119.2 (4)
C12—C11—C1	120.4 (3)	C56—C51—C5	120.4 (3)
C16—C11—C1	120.0 (3)	C52—C51—C5	120.3 (3)
C13—C12—C11	119.9 (4)	C53—C52—C51	120.1 (4)
C13—C12—H12	120.0	C53—C52—H52	119.9
C11—C12—H12	120.0	C51—C52—H52	119.9
C14—C13—C12	120.1 (4)	C52—C53—C54	120.4 (4)
C14—C13—H13	120.0	C52—C53—H53	119.8
C12—C13—H13	120.0	C54—C53—H53	119.8
C13—C14—C15	120.4 (4)	C53—C54—C55	119.9 (4)
C13—C14—H14	119.8	C53—C54—H54	120.1
C15—C14—H14	119.8	C55—C54—H54	120.1
C14—C15—C16	120.0 (4)	C56—C55—C54	119.9 (4)
C14—C15—H15	120.0	C56—C55—H55	120.0
C16—C15—H15	120.0	C54—C55—H55	120.0
C15—C16—C11	120.1 (4)	C55—C56—C51	120.4 (4)
C15—C16—H16	120.0	C55—C56—H56	119.8
C11—C16—H16	120.0	C51—C56—H56	119.8
C22—C21—C26	119.1 (4)	C62—C61—C66	119.2 (4)
C22—C21—P1	122.2 (3)	C62—C61—P2	122.5 (3)
C26—C21—P1	118.6 (3)	C66—C61—P2	118.3 (3)
C23—C22—C21	120.3 (4)	C63—C62—C61	120.1 (4)
C23—C22—H22	119.8	C63—C62—H62	119.9
C21—C22—H22	119.8	C61—C62—H62	119.9
C24—C23—C22	120.1 (5)	C64—C63—C62	120.4 (4)
C24—C23—H23	120.0	C64—C63—H63	119.8
C22—C23—H23	120.0	C62—C63—H63	119.8
C25—C24—C23	120.1 (4)	C63—C64—C65	120.0 (4)
C25—C24—H24	119.9	C63—C64—H64	120.0
C23—C24—H24	119.9	C65—C64—H64	120.0
C24—C25—C26	120.2 (4)	C66—C65—C64	120.0 (4)
C24—C25—H25	119.9	C66—C65—H65	120.0
C26—C25—H25	119.9	C64—C65—H65	120.0
C25—C26—C21	120.1 (4)	C65—C66—C61	120.4 (4)
C25—C26—H26	119.9	C65—C66—H66	119.8
C21—C26—H26	119.9	C61—C66—H66	119.8
C36—C31—C32	119.0 (4)	C76—C71—C72	118.8 (4)
C36—C31—P1	120.2 (3)	C76—C71—P2	121.9 (3)
C32—C31—P1	120.5 (3)	C72—C71—P2	118.8 (3)
C33—C32—C31	120.3 (4)	C73—C72—C71	120.8 (4)
C33—C32—H32	119.8	C73—C72—H72	119.6
C31—C32—H32	119.8	C71—C72—H72	119.6
C34—C33—C32	119.9 (4)	C72—C73—C74	119.4 (5)
C34—C33—H33	120.1	C72—C73—H73	120.3
C32—C33—H33	120.1	C74—C73—H73	120.3
C33—C34—C35	120.2 (4)	C75—C74—C73	120.4 (4)
C33—C34—H34	119.9	C75—C74—H74	119.8
C35—C34—H34	119.9	C73—C74—H74	119.8
C36—C35—C34	120.2 (4)	C74—C75—C76	120.5 (5)
C36—C35—H35	119.9	C74—C75—H75	119.7
C34—C35—H35	119.9	C76—C75—H75	119.7
C35—C36—C31	120.4 (4)	C75—C76—C71	120.0 (4)
C35—C36—H36	119.8	C75—C76—H76	120.0
C31—C36—H36	119.8	C71—C76—H76	120.0
C42—C41—C46	119.0 (4)	C82—C81—C86	119.1 (4)
C42—C41—P1	118.5 (3)	C82—C81—P2	119.9 (3)
C46—C41—P1	122.4 (3)	C86—C81—P2	120.9 (3)
C43—C42—C41	120.6 (4)	C83—C82—C81	120.4 (4)
C43—C42—H42	119.7	C83—C82—H82	119.8
C41—C42—H42	119.7	C81—C82—H82	119.8
C44—C43—C42	120.2 (4)	C84—C83—C82	120.2 (4)
C44—C43—H43	119.9	C84—C83—H83	119.9
C42—C43—H43	119.9	C82—C83—H83	119.9
C45—C44—C43	119.9 (4)	C83—C84—C85	119.9 (4)
C45—C44—H44	120.0	C83—C84—H84	120.1
C43—C44—H44	120.0	C85—C84—H84	120.1
C44—C45—C46	120.3 (4)	C84—C85—C86	120.1 (4)
C44—C45—H45	119.8	C84—C85—H85	120.0
C46—C45—H45	119.8	C86—C85—H85	120.0
C45—C46—C41	119.9 (4)	C85—C86—C81	120.4 (4)
C45—C46—H46	120.0	C85—C86—H86	119.8
C41—C46—H46	120.0	C81—C86—H86	119.8
			
O2—Re1—P1—C21	−173.65 (17)	O4—Re2—P2—C71	85.74 (17)
O1—Re1—P1—C21	19.68 (15)	O3—Re2—P2—C71	−82.68 (15)
N1—Re1—P1—C21	92.87 (16)	N3—Re2—P2—C71	−9.29 (16)
Br1—Re1—P1—C21	−69.61 (14)	Br4—Re2—P2—C71	−171.74 (14)
O2—Re1—P1—C31	65.38 (16)	O4—Re2—P2—C61	−154.11 (17)
O1—Re1—P1—C31	−101.29 (15)	O3—Re2—P2—C61	37.47 (16)
N1—Re1—P1—C31	−28.11 (15)	N3—Re2—P2—C61	110.86 (16)
Br1—Re1—P1—C31	169.42 (13)	Br4—Re2—P2—C61	−51.59 (14)
O2—Re1—P1—C41	−49.88 (16)	O4—Re2—P2—C81	−30.82 (16)
O1—Re1—P1—C41	143.44 (15)	O3—Re2—P2—C81	160.76 (14)
N1—Re1—P1—C41	−143.37 (16)	N3—Re2—P2—C81	−125.85 (15)
Br1—Re1—P1—C41	54.15 (13)	Br4—Re2—P2—C81	71.70 (13)
O2—Re1—O1—C1	45.1 (5)	O4—Re2—O3—C5	46.6 (5)
N1—Re1—O1—C1	−4.7 (2)	N3—Re2—O3—C5	−5.5 (3)
P1—Re1—O1—C1	91.7 (2)	P2—Re2—O3—C5	88.1 (2)
Br1—Re1—O1—C1	−176.1 (2)	Br4—Re2—O3—C5	−177.7 (2)
Br2—Re1—O1—C1	−87.8 (2)	Br3—Re2—O3—C5	−89.3 (2)
O2—Re1—N1—C2	22.8 (4)	O4—Re2—N3—C6	19.8 (4)
O1—Re1—N1—C2	−171.2 (4)	O3—Re2—N3—C6	−174.0 (4)
P1—Re1—N1—C2	106.6 (4)	P2—Re2—N3—C6	104.2 (4)
Br1—Re1—N1—C2	−143.4 (3)	Br4—Re2—N3—C6	−148.5 (3)
Br2—Re1—N1—C2	−78.5 (4)	Br3—Re2—N3—C6	−80.9 (4)
O2—Re1—N1—N2	−161.3 (3)	O4—Re2—N3—N4	−160.7 (3)
O1—Re1—N1—N2	4.7 (2)	O3—Re2—N3—N4	5.5 (2)
P1—Re1—N1—N2	−77.5 (2)	P2—Re2—N3—N4	−76.3 (2)
Br1—Re1—N1—N2	32.5 (4)	Br4—Re2—N3—N4	30.9 (5)
Br2—Re1—N1—N2	97.3 (2)	Br3—Re2—N3—N4	98.5 (2)
C2—N1—N2—C1	172.6 (3)	C6—N3—N4—C5	174.9 (4)
Re1—N1—N2—C1	−4.0 (4)	Re2—N3—N4—C5	−4.7 (4)
N1—N2—C1—O1	−0.1 (5)	N3—N4—C5—O3	0.0 (5)
N1—N2—C1—C11	177.7 (3)	N3—N4—C5—C51	179.4 (3)
Re1—O1—C1—N2	4.5 (5)	Re2—O3—C5—N4	5.1 (5)
Re1—O1—C1—C11	−173.4 (2)	Re2—O3—C5—C51	−174.3 (2)
N2—N1—C2—C4	−178.6 (4)	N4—N3—C6—C7	4.8 (6)
Re1—N1—C2—C4	−2.6 (6)	Re2—N3—C6—C7	−175.8 (3)
N2—N1—C2—C3	1.7 (6)	N4—N3—C6—C8	−176.4 (4)
Re1—N1—C2—C3	177.6 (3)	Re2—N3—C6—C8	3.0 (7)
N2—C1—C11—C12	14.6 (5)	N4—C5—C51—C56	−173.1 (4)
O1—C1—C11—C12	−167.5 (3)	O3—C5—C51—C56	6.3 (5)
N2—C1—C11—C16	−163.4 (4)	N4—C5—C51—C52	6.3 (6)
O1—C1—C11—C16	14.6 (5)	O3—C5—C51—C52	−174.3 (3)
C16—C11—C12—C13	0.4 (6)	C56—C51—C52—C53	−0.1 (6)
C1—C11—C12—C13	−177.6 (4)	C5—C51—C52—C53	−179.5 (4)
C11—C12—C13—C14	−0.4 (6)	C51—C52—C53—C54	0.8 (6)
C12—C13—C14—C15	0.3 (7)	C52—C53—C54—C55	−0.8 (7)
C13—C14—C15—C16	−0.2 (7)	C53—C54—C55—C56	0.2 (7)
C14—C15—C16—C11	0.2 (7)	C54—C55—C56—C51	0.5 (7)
C12—C11—C16—C15	−0.3 (6)	C52—C51—C56—C55	−0.5 (6)
C1—C11—C16—C15	177.7 (4)	C5—C51—C56—C55	178.9 (4)
C31—P1—C21—C22	−13.9 (4)	C71—P2—C61—C62	133.1 (3)
C41—P1—C21—C22	96.2 (4)	C81—P2—C61—C62	−116.6 (3)
Re1—P1—C21—C22	−135.4 (3)	Re2—P2—C61—C62	10.6 (4)
C31—P1—C21—C26	168.8 (3)	C71—P2—C61—C66	−48.2 (4)
C41—P1—C21—C26	−81.1 (3)	C81—P2—C61—C66	62.0 (4)
Re1—P1—C21—C26	47.3 (3)	Re2—P2—C61—C66	−170.8 (3)
C26—C21—C22—C23	−1.5 (7)	C66—C61—C62—C63	0.6 (6)
P1—C21—C22—C23	−178.7 (4)	P2—C61—C62—C63	179.2 (3)
C21—C22—C23—C24	0.4 (8)	C61—C62—C63—C64	0.0 (6)
C22—C23—C24—C25	0.9 (8)	C62—C63—C64—C65	−0.7 (6)
C23—C24—C25—C26	−1.2 (7)	C63—C64—C65—C66	0.7 (7)
C24—C25—C26—C21	0.1 (7)	C64—C65—C66—C61	0.0 (7)
C22—C21—C26—C25	1.2 (6)	C62—C61—C66—C65	−0.6 (6)
P1—C21—C26—C25	178.5 (3)	P2—C61—C66—C65	−179.3 (3)
C21—P1—C31—C36	−52.4 (4)	C61—P2—C71—C76	−31.9 (4)
C41—P1—C31—C36	−163.3 (3)	C81—P2—C71—C76	−141.4 (3)
Re1—P1—C31—C36	74.7 (3)	Re2—P2—C71—C76	97.5 (3)
C21—P1—C31—C32	134.6 (3)	C61—P2—C71—C72	156.3 (3)
C41—P1—C31—C32	23.7 (4)	C81—P2—C71—C72	46.8 (4)
Re1—P1—C31—C32	−98.3 (3)	Re2—P2—C71—C72	−74.3 (3)
C36—C31—C32—C33	0.0 (6)	C76—C71—C72—C73	1.7 (7)
P1—C31—C32—C33	173.1 (4)	P2—C71—C72—C73	173.7 (4)
C31—C32—C33—C34	0.8 (7)	C71—C72—C73—C74	−1.1 (7)
C32—C33—C34—C35	−0.9 (8)	C72—C73—C74—C75	0.0 (8)
C33—C34—C35—C36	0.0 (7)	C73—C74—C75—C76	0.6 (8)
C34—C35—C36—C31	0.8 (7)	C74—C75—C76—C71	0.0 (7)
C32—C31—C36—C35	−0.8 (6)	C72—C71—C76—C75	−1.1 (6)
P1—C31—C36—C35	−173.9 (3)	P2—C71—C76—C75	−172.9 (4)
C21—P1—C41—C42	−45.5 (4)	C71—P2—C81—C82	−149.4 (3)
C31—P1—C41—C42	66.8 (4)	C61—P2—C81—C82	101.1 (3)
Re1—P1—C41—C42	−175.6 (3)	Re2—P2—C81—C82	−30.6 (3)
C21—P1—C41—C46	138.9 (3)	C71—P2—C81—C86	33.0 (4)
C31—P1—C41—C46	−108.8 (3)	C61—P2—C81—C86	−76.5 (3)
Re1—P1—C41—C46	8.8 (4)	Re2—P2—C81—C86	151.8 (3)
C46—C41—C42—C43	0.4 (7)	C86—C81—C82—C83	0.4 (6)
P1—C41—C42—C43	−175.3 (4)	P2—C81—C82—C83	−177.2 (3)
C41—C42—C43—C44	0.2 (8)	C81—C82—C83—C84	−0.3 (6)
C42—C43—C44—C45	−0.7 (8)	C82—C83—C84—C85	0.2 (6)
C43—C44—C45—C46	0.8 (7)	C83—C84—C85—C86	−0.2 (6)
C44—C45—C46—C41	−0.2 (7)	C84—C85—C86—C81	0.3 (6)
C42—C41—C46—C45	−0.4 (6)	C82—C81—C86—C85	−0.4 (6)
P1—C41—C46—C45	175.2 (3)	P2—C81—C86—C85	177.2 (3)

### Hydrogen-bond geometry (Å, °)

**Table:**

*D*—H···*A*	*D*—H	H···*A*	*D*···*A*	*D*—H···*A*
C22—H22···Br3	0.95	2.94	3.819 (5)	154.
C85—H85···Br2^i^	0.95	2.92	3.587 (4)	128.

Symmetry codes: (i) *x*−1, −*y*+1/2, *z*−1/2.
